# Mr. Norris' Account of Professor Porson's Death

**Published:** 1808-11

**Authors:** William Norris

**Affiliations:** Old Jewry


### To Dr. ADAMS.

Dear Sir,

In compliance with your request to be informed of the disease which occasioned the death of that extraordinary literary genius, the late Professor Porson, and of the appearances on dissection, I herewith inclose the best account that I am able to give. I lament that the history of his illness is necessarily very imperfect, because he had not been visited professionally by any medical person, until a short period before his death.

On Friday, September 16, I called to speak to him about a book, and was shocked at his generally diseased appearance. I asked if he was not sensible of much indisposition; to which he replied, that he had been ill for some time of *ague and fever,* but that he now thought himself better.

His countenance was pale; his skin was hot; his pulse quick and feeble; and his tongue white. I told him that I supposed his reason for using the term ague, was his having had cold fits succeeded by heat, to which he assented; that these symptoms were common to almost all fevers, however excited; that he was at that moment very seriously ill from a cause intirely different from what he imagined; and I concluded by begging him to send for my friend Mr. Upton, who was just at hand, or for some physician of his own acquaintance. To this, however, he would not consent, as he said he was now better; but I so far prevailed as to obtain his promise to do what I desired the next morning, if he should not continue to improve. To a message which I, the next day, sent, he returned for answer that he was better.

I heard nothing more of him until the evening of Tuesday the 20th, when Mr. Savage, the under librarian, came for me, and said that Mr. Porson had had a fit, as he believed, of epilepsy, and that he was much alarmed about him. I found Mr. Porson sitting up, and staring about as if surprised. The only answer that I could obtain to any question was, Well! How? What? and he appeared to be utterly incapable of reasoning or of comprehending what was said to him.

In this state he was put to bed, and I sent immediate notice of his situation to his brother-in-law, Mr. Perry, who very soon arrived, and who continued to the last to pay him the kindest attention, with the most affectionate solicitude.

A clyster

A clyster was injected, and he was with difficulty made to swallow a pill containing two grains of calomel; and some hours afterwards, a draft of infusion of senna and Epsom salt. These procured two or three copious motions, after which he brightened up, and for a short time seemed much relieved.

Dr. Babington and Mr. Upton now saw him, when stupor had again returned, accompanied by such a state of general debility as to preclude all idea of farther evacuation. Blisters and sinapisms were applied, which produced transient relief; and it was endeavoured to support his strength by wine and cordial medicines, of which, however, very little was swallowed. He continued, with a few slight and short appearances of amendment, to grow weaker until Sunday night, when he died; having gradually lost the power of speech and sight, so that two days before his death, his eyes were perfectly insensibe to the light of a candle.

The following account was given to Mr. Perry, signed by Dr. Babington, Sir W. Blizard, Mr. Thomas Blizard, Mr. Upton, and myself.

" On examining the body of Professor Porson, we observed the following appearances.

" The body was emaciated.

" The dura mater did not exhibit any unusual appearance.

" Under the tunica arachnoïdes a clear fluid was seen to be generally diffused over the surface of the brain; and upon separating the pia mater, lymph, to the quantity of about an ounce, issued from between the convolutions of the brain.

" The brain was of an unusually firm texture, its cortical part was of a lighter colour, and its medullary part was less white than is common.

" The ventricles did not seem to contain more than one ounce of lymph; but upon removing the whole of the brain, at least an ounce and half more lymph remained at the basis of the skull.

" The abdominal viscera did not present any thing particularly worthy of notice. The substance of the intestines, indeed, was unusually thick, as was that of the bladder; there was an adhesion of the omentum to the liver, and several more between it and the diaphragm; and in its peritoneal covering there was a small ossification. The pylorus was very narrow, but without disease. To none of these circumstances do we attach any consequence, as they do not appear to have had any share in producing death.

" The

"The heart was sound, and the pericardium contained the usual quantity of lymph.

"The left lung had many adhesions to the pleura, and bore visible marks of former inflammation. The right lung was in a perfectly sound state.

"From a due consideration of these circumstances, and of the symptoms observed during the short period of his confinement, as well as of what we knew to have been his usual sedentery mode of living, we are of opinion that the effused lymph in and upon the brain, which we believe to have been the effect of recent inflammation, was the immediate cause of his death. It may also be observed that his health had been in a declining state during some months, so as to have been visible to his friends."

It is very clear that, during the indisposition, which he called ague and fever, a slow inflammatory action was going on within the head, the result of which was the effusion above noticed. The first effect of compression from this cause that was noticed, was on Monday the 19th, on which day he walked from the Old Jewry to the west end of the town, when he fell in the street, and was taken in a state of insensibility into a neighbouring house, where he remained all night. After this he did not *perfectly* recover his intellects.

WILLIAM NORRIS.

*Old Jewry, October* 7, 1808.

